# Clinical efficacy and pain follow-up after arthroscopic discoid meniscoplasty of the knee: a retrospective study

**DOI:** 10.1186/s12891-024-07683-9

**Published:** 2024-07-22

**Authors:** Jingri Jin, Xiaodan Xu, Mingjun Piao, Weize Sun, Guangzhi Pan, Yanqun Liu

**Affiliations:** 1 Department of Joint Surgery, The Affiliated Hospital of Yanbian University, 1327 Juzi Street, Yanji City, Jilin Province 133000 P.R. China; 2 Department of Operating Room, The Affiliated Hospital of Yanbian University, Yanji City, Jilin Province China

**Keywords:** Arthroscopty, Meniscoplasty, Discoid meniscus, Clinical efficacy, Postoperative effect

## Abstract

**Background:**

To assess the viability and efficiency of performing arthroscopic meniscoplasty in treating discoid meniscus (DM) in the knee joint.

**Methods:**

A total of 29 patients diagnosed with symptomatic lateral DM between October 2014 and December 2019 were included in the study. Among them, 7 patients with intact DM underwent arthroscopic discoid meniscoplasty (group A), while 22 patients with torn DM received arthroscopic DM plasty along with repair and suturing (group B). Both Visual Analog Scale (VAS) score and Lysholm score assessments were conducted preoperatively and postoperatively.

**Result:**

The favorable and acceptable outcome rate was 85.71% in group A and 95.45% in group B (*P* > 0.05). The VAS scores post-operatively at each follow-up time point were consistently lower compared to pre-operative values, while the Lysholm knee function scores showed improvement. There were no significant differences in VAS score and Lysholm score between group A and group B at different stages (*P* > 0.05). Both surgical techniques (discoid meniscoplasty and discoid meniscoplasty combined with repair and suture) showed significant improvement in postoperative VAS score and Lysholm score, but there was no difference in outcomes between the two groups.

**Conclusion:**

Overall, the two surgical techniques studied in this study (discoid meniscoplasty and discoid meniscoplasty combined with repair and suture) produced similar results in terms of pain reduction and improved knee function.

## Introduction

The meniscus, located between the femoral condyle and the tibial plateau, has a crescent shape and is comprised of 70% water and 30% organic material. Within the organic content, 75% is collagen, predominantly type I collagen, arranged in a ring formation [1]. Discoid meniscus (DM) is a rare abnormality characterized by a larger and thicker meniscus compared to the typical structure, with a prevalence of less than 5% in the general population [2]. The enlarged size of the DM can restrict the blood supply to the joints, making it more prone to injuries and tears during intense physical activity [3]. The existence of DM was first documented by Young in 1889. Subsequent autopsies by Noble and Hamblen in 1975 revealed that around 5% of individuals had a DM, mostly situated on the lateral side. According to local statistics, lateral DM represents 25.3% of cases, while medial cases are much rarer at 0.57% [4].

Currently, the definitive cause of DM remains unclear, lacking a consensus and requiring further investigation. In 1948, Simillie postulated that DM arises from the retention and inadequate resorption of the central portion of the meniscus during prenatal development [5]. He posited that the DM represents a congenital “persistent fetal condition.” The meniscus originates from mesodermal cells of the femur and tibia. In the prenatal period, the cartilaginous plates within and around the knee joint merge to form a complete cartilaginous plate partition between the plate bones, assuming a discoid shape. As fetal development progresses, accompanied by the maturation of the cruciate ligament, the central portion of the cartilaginous plate is gradually reabsorbed, culminating in the formation of a typical meniscus. However, should the physiological reabsorption process be incomplete for any reason, a DM will ensue. Barnes et al. documented a case of a DM in a patient as young as 6 months old, lending support to the theory of congenital developmental irregularities.

Although the formation mechanism of DM is not fully understood, the pathophysiology, diagnosis and treatment strategies of DM have been extensively investigated. Studies have shown that the abnormal shape of DM may be related to genetic factors [6], and is prone to meniscus tears, resulting in knee pain and dysfunction. Magnetic resonance imaging (MRI) is currently the primary tool for diagnosing DM and is able to clearly show the shape of the meniscus and any potential tears [7]. In addition, arthroscopy is widely used to confirm diagnosis and develop treatment plans [8]. For patients with DM with obvious symptoms, surgical intervention is the main treatment, including meniscoplasty and partial meniscectomy [9], while postoperative rehabilitation training is essential to restore knee function. Despite the wealth of information provided by the above studies, there are still many areas that need further exploration. At present, there is a lack of data on the long-term efficacy of surgical treatment of DM. Future studies should focus on follow-up to evaluate the long-term effects and complications after surgery [10]. The effectiveness of non-surgical treatment for patients with mild or asymptomatic DM has not been adequately studied. Although some studies have suggested that the formation of DM may be related to genetic factors, the specific genetic mechanism and the influence of environmental factors still need to be further explored. In addition, different patients have different conditions and needs, and research on individualized treatment plans can improve treatment effect and patient satisfaction, which requires more clinical trials and big data analysis [11]. By further studying these areas, the management of DM can be more comprehensively understood and the treatment effect and quality of life of patients can be improved.

From October 2014 to December 2019, the Department of Arthroscopy at the affiliated Hospital of Yanbian University treated 29 patients with symptomatic DM. Surgical interventions included simple meniscoplasty and meniscoplasty combined with repair and suturing. The study conducted an analysis of the clinical outcomes and postoperative pain following arthroscopic treatment for DM injuries. The aim of this study was to evaluate the long-term efficacy of different treatments for patients with DM, and to explore the potential advantages of individualized treatment regimens. It is hypothesized that the knee function and quality of life of patients with DM will be significantly improved after surgical intervention, and the long-term curative effect is better than that of non-surgical treatment.

## Methods

### General information

From October 2014 to December 2019, our department treated a total of 29 patients presenting symptoms related to the lateral DM. The inclusion criteria were as follows: patients diagnosed with unilateral knee lateral DM injury, and patients with clear indications for surgical treatment, such as persistent pain, locking, and mechanical symptoms unresponsive to conservative management [12, 13]. The exclusion criteria included patients diagnosed with medial DM injury, those with any type of tumor, those with acute infection, those who had received other surgical treatments in the past 3 months, patients with advanced age, osteoarthritis, or other traumatic injuries such as ACL rupture. Among them, 7 patients belonged to group A, which consisted of those who underwent arthroscopic discoid meniscoplasty, while the remaining 22 patients were in group B, which included those who underwent a combination of arthroscopic discoid meniscoplasty with repair and suturing. The Department of Arthropathy of the affiliated Hospital of Yanbian University Ethical Committee approved the study and informed consent was obtained from each participant before testing.

### Operation procedure

Under either spinal or general anesthesia, the patient was positioned supine, with an air bag tourniquet (pressure 280mmHg) secured at the base of the affected leg. After preparing the disinfection sheet and setting up the arthroscopic instruments, the blood tourniquet was released and reinflated. With the knee flexed at 90°, the anterolateral approach was initiated by following the lateral border of the patellar ligament to the inferior angle of the patella, enabling a comprehensive exploration and clearance of the knee joint cavity, addressing hyperplastic synovium and fat pad. Special attention was paid to assessing the lateral DM for any associated injuries, identifying the location and severity of any damage, and evaluating the overall tissue status, including meniscus thickness and articular cartilage condition. Subsequently, the anterior medial approach was established, tracing along the medial edge of the patellar ligament to the triangular region delimited by the bony prominences of the medial femoral condyle and the medial tibial plateau as the primary surgical access point.

Patients in group A underwent treatment using meniscus basket forceps and Dyonics enhanced planing tool head from SmithNephew, USA. The lateral DM was partially removed, planed, attracted, and polished along the medial edge. Following reshaping to a satisfactory form, the bipolar meniscectomy probe (SmithNephew, USA) was employed to trim along the medial edge of the meniscus and underwent “slope” treatment. The meniscus was shaped back to a “quasi-normal meniscus” form, with a width ranging from 6 to 8 mm. The standard procedure for DM formation in group B matched that of group A. In cases of a horizontal meniscus tear, the inactive tissue at the tear site was excised using a planing knife. Subsequent to improved freshness treatment, the disposable meniscus repair system from DePuyMitek, USA implemented the Fast-Fix Allinside technique for vertical or horizontal sutures placed at the posterior horn, body, or anterior horn of the meniscus. Utilizing two cannula needles paired with PDS II thread facilitated suture tool construction, with the option to apply the Out-side-in technique for vertical or horizontal suturing. Before knot fixation, replacing the PDS II line with an irretrievable thread was carried out. Under arthroscopic monitoring, the knot was secured outside the joint with suitable tension, ensuring its concealment between the deep subcutaneous tissue and joint capsule, thus accomplishing the goals of meniscus repair, suture, and fixation.

### Postoperative treatment

Following the surgery, both groups had their affected knees wrapped with elastic bandages for a week and secured with digital chuck adjustable braces for 2–3 weeks. Patients were instructed to engage in isometric and isotonic contraction of the quadriceps femoris starting from the day after the operation, and the drainage tube was removed on the second day post-surgery. Notably, for group B, after the drainage tube removal, bedside passive knee flexion exercises were initiated once or twice daily. Normal passive knee joint flexion was achieved within 2 weeks, followed by active flexion and extension exercises after another 2 weeks. Weight-bearing standing and walking exercises could be fully performed after 4–6 weeks, with gradual resumption of physical activities and work after 6–8 weeks. In contrast, patients in group A could start active and passive flexion and extension exercises of the knee joint, as well as weight-bearing walking exercises in a progressive manner after drainage tube removal, with the possibility of resuming sports and work after 4–6 weeks. The braces should be removed during knee flexion and extension exercises. Applying ice to the affected knee for 15 min following each 10–15 min of practice (optimal ice-water ratio) is recommended.

### Evaluation of curative effect

Postoperative efficacy was evaluated by Ikeuchi grade [14], the score of which was obtained through regular follow-up by the outpatient visit. In addition, the assessment of knee joint pain and function utilized the VAS score [15] and Lysholm score [16] criteria pre-surgery and post-surgery. The effectiveness of the surgery was determined based on the Lysholm score assessment. Follow-up evaluations were conducted at 3 months, 6 months, 1 year, 3 years, and 5 years postoperatively to monitor knee joint stability, pain levels, swelling, and gait abnormalities.

### Statistical analyses

The data underwent analysis using SPSS 22.0 software (IBM Corp., USA). The mean ± standard deviation (SD) was utilized to present the measurement data before and after the operation. Categorical data were expressed as [n (%)]. Group differences were assessed through the chi-square tests or Fisher’s exact test. In the comparison of measurement data of the same type of operation before and after the final follow-up, the paired t-test was employed. Grade data were analyzed using the Mann-Whitney rank sum test. Comparison of measurement data from two different methods at the same follow-up time was conducted with the two-independent-sample *t*-test. A significance level of α = 0.05 (*P* < 0.05) was set for statistical interpretation of differences.

## Results

The demographic distribution of the patients included 19 Korean individuals, 6 from the Han nationality, and 4 from the Manchu ethnicity. The gender distribution comprised 18 males (62.07%) and 11 females (37.93%), with the majority being amateur athletes, resulting in a male-to-female ratio of 18: 11. The patients involved in this study presented symptoms related to the lateral DM. These symptoms included recurrent knee joint swelling and pain, lateral knee joint pain, evident joint instability, limited flexion and extension of the knee, and joint catching or locking. The average age of the patients was 40 years, ranging from 15 to 53 years. In group B, the patients presented with various conditions such as horizontal tear in 13 cases, longitudinal tear in the red-red area in 7 cases, complex tear in the red-white area (bucket handle + radial tear) in 2 cases, knee sprain in 4 cases, fall injury in 5 cases, and sports injury in 11 cases. The rest of the patients did not have a clear history of significant trauma, some only experiencing mild trauma. Common symptoms among all patients included recurrent knee joint swelling and pain, lateral knee joint pain, evident joint instability in 20 cases, limited flexion and extension in 17 cases (including 2 cases of knee joint locking). The average time from symptom onset to surgical intervention was 8 weeks, ranging from 1 to 40 weeks. During arthroscopic examination, 21 cases were identified with a complete DM and 8 cases with an incomplete meniscus. The articular cartilage injury severity, graded according to the Outerbridge classification, was below grade II in all patients, primarily presenting as articular chondromalacia and rough changes (Table 1).

**Table 1:** Demographic and clinical characteristics

Characteristics	GroupA (*n* = 7)	GroupB (*n* = 22)	Total (*n* = 29)
Age (years)	38.4 ± 5.1	39.2 ± 4.8	40 (range 15–53)
Ethnicity [n (%)]			
Korean	5 (71.43%)	14 (63.64%)	19 (48.28%)
Han	1 (14.29%)	5 (22.73%)	6 (20.69%)
Manchu	1 (14.29%)	3 (13.64%)	4 (13.79%)
Gender [n (%)]			
Male	4 (57.14%)	14 (63.64%)	18 (62.07%)
Female	3 (42.86%)	8 (36.36%)	11 (37.93%)
Common Symptoms [n (%)]			
Recurrent knee joint swelling and pain	7 (100.00%)	22 (100.00%)	29 (100.00%)
Lateral knee joint pain	7 (100.00%)	22 (100.00%)	29 (100.00%)
Joint instability	5 (71.43%)	15 (68.18%)	20 (68.97%)
Limited flexion and extension	4 (57.14%)	13 (59.09%)	17 (58.62%)
Knee joint locking	0 (0.00%)	2 (9.09%)	2 (6.90%)
Arthroscopic Findings [n (%)]			
Complete DM	5 (71.43%)	16 (72.73%)	21 (72.41%)
Incomplete meniscus	2 (28.57%)	6 (27.27%)	8 (27.59%)
Articular Cartilage Injury (Outbridge ≤ II)	7 (100.00%)	22 (100.00%)	29 (100.00%)
Symptom Duration (weeks)	8 (range 1–40)	8 (range 1–40)	8 (range 1–40)

All patients were monitored for a minimum of 5 years post-operation. According to Ikeuchi grade: Excellent: No symptoms, full recovery to normal activities, normal knee joint function without any discomfort. Good: Most symptoms have disappeared, capable of performing most daily activities, knee joint function is near normal, occasional mild discomfort that does not affect normal life. Fair: Symptoms have improved but some discomfort and functional limitations persist, daily activities are somewhat affected, occasional need for medication or assistive devices. Poor: Symptoms persist with significant discomfort and functional limitations, daily activities are substantially affected, frequent need for medication or assistive devices, following up operation, the outcomes for group A were deemed excellent in 5 instances, good in 1, fair in 1, and no cases were categorized as poor. The combined rate of excellent and good outcomes in group A stood at 85.71%. Specifically in group B, there were 18 cases of excellent outcomes, 3 cases of good outcomes, 1 case of fair outcome, and none classified as poor, resulting in an overall rate of 95.45% for excellent and good outcomes. There was no significant difference between the two groups (*P* > 0.05) (Table 2).

**Table 2:** Comparison of postoperative excellent and good rates between the two groups

Curative effect	Group A	Group B	*P* value
Excellent	5	18	0.53
Good	1	3	
Fair	1	1	
Excellence rate	85.71%	95.45%	

**Fig. 1:**
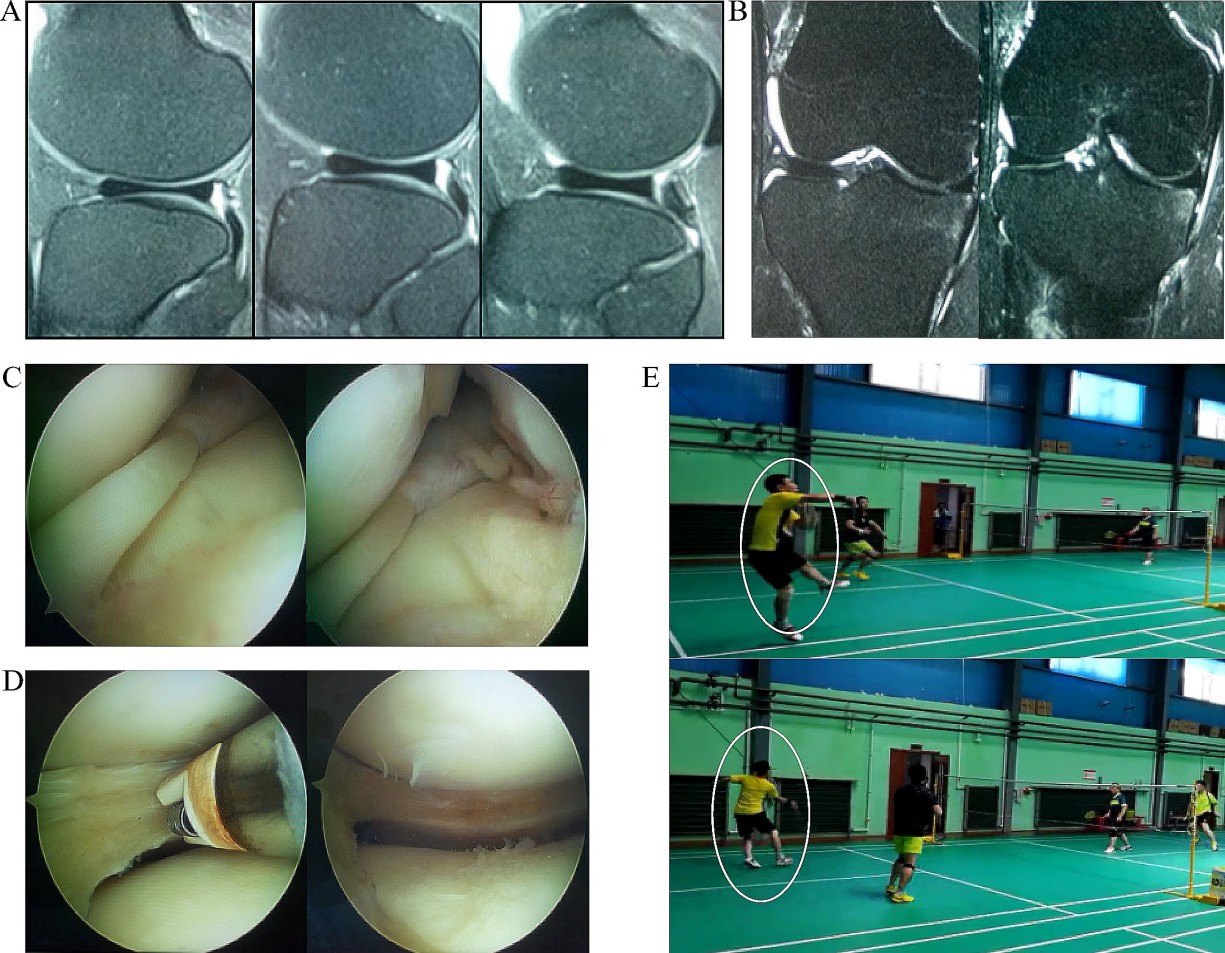
Arthroscopic simple DM plasty for the treatment of lateral DM (male, 52 years old, right knee pain and movement limitation caused by fall during exercise for more than one month. Amateur badminton player). (**A**) MRI showed that the anterior and posterior corners of more than 3 consecutive sagittal planes were connected. (**B**) The transverse diameter of lateral meniscus in MRI coronal plane exceeded 20% of the widest transverse diameter of tibial plateau, but the medial edge did not reach the intercondylar eminence. (**C**) The Watanbe classification of lateral DM is incomplete DM. (**D**) The part of DM is resected, trimmed and formed under arthroscopy and no meniscus injury was found, moreover, the thickness of meniscus was about 4 mm and the medial edge of meniscus was treated with plasma radiofrequency knife. (**E**) The patients participating in the badminton match

**Fig. 2:**
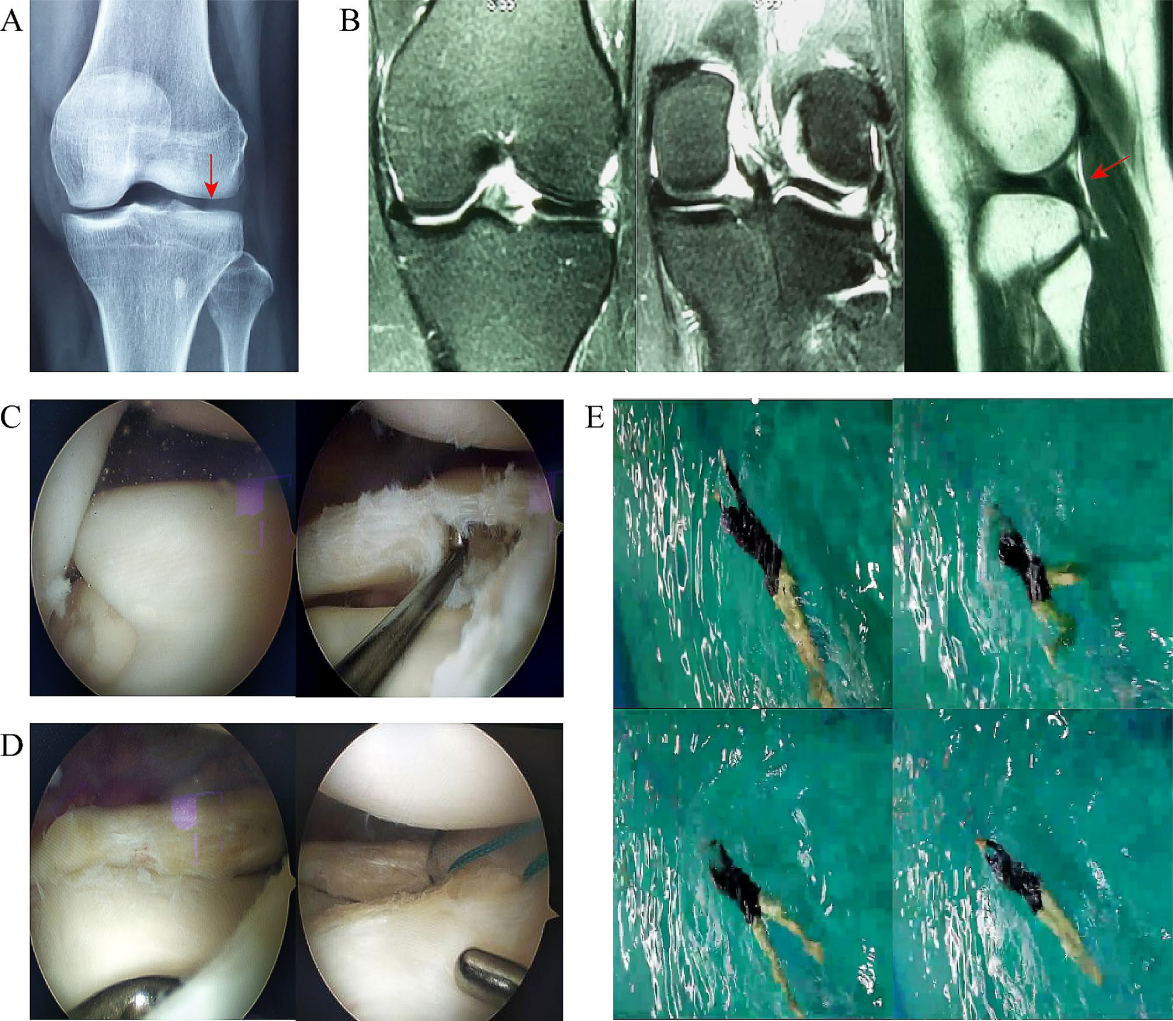
Arthroscopic DM plasty combined with repair and suture in the treatment of lateral DM of the knee (female, 47 years old, without obvious inducement, pain in the left knee and limitation of movement for 1 month. Amateur swimmer). (**A**) Anterior X-ray film of the knee joint showed that the distal femoral condyle was obviously flattened. (**B**) The transverse diameter of lateral meniscus on MRI coronal plane before exceeded 20% of the widest transverse diameter of tibial plateau, but the medial edge reached intercondylar eminence, suggesting complete DM. (**C**) Sagittal DM with horizontal tear arthroscopy further confirmed that the Watanbe classification of lateral DM was complete and was partially resected, trimmed and resected. (**D**) It can be seen that the central area of the meniscus was obviously torn horizontally under arthroscopy, and the medial edge of the meniscus was repaired with a plasma radio frequency knife, and the thickness of the meniscus was about 4 mm, and the meniscus in the tearing area was sutured and fixed with 3 needles using Out-side-in technique. (**E**) The picture shows the decomposition of the breaststroke shown by the patient

We reported two representative cases and showed detailed case illustrations (Figs. 1 and 2). According to the cases, there were no reported complications such as incision infection, knee joint stiffness, knee joint bounce recurrence, or meniscus tear within this cohort before the conclusion of the study.

Using the VAS scores and the Lysholm knee scoring scale, there were significant differences in the different time periods in each group (*P* < 0.05). Considerable improvement was observed after surgery in each group; however, there was no significant difference in the VAS scores and the Lysholm knee scoring scale between the groups at the same stage (*P* > 0.05) (Tables 3 and 4). The results showed that arthroscopic discoid meniscoplasty could relieve pain after 3 months, with no recurrence within 5 years. Arthroscopic discoid meniscoplasty of the knee is a safe and effective surgical method that could significantly reduce pain, moreover, arthroscopic discoid meniscoplasty and arthroscopic DM plasty along with repair and suturing could effectively improve knee joint function.

**Table 3:** Comparison of VAS scores between Group A and Group B

Group	Cases	Pre-op	3 months	6 months	1 year	3 years	5 years	*P* value
Group A	7	8.0 ± 0.82	5.71 ± 0.95	1.86 ± 0.69	1.57 ± 0.53	1.14 ± 0.38	0.86 ± 0.38	0.001
Group B	22	8.32 ± 0.72	6.18 ± 0.80	2.45 ± 0.91	1.64 ± 0.58	1.13 ± 0.35	0.91 ± 0.61	0.001
t		0.99	1.29	1.59	0.26	0.04	0.21	
*P*		0.33	0.21	0.12	0.8	0.97	0.83	

*Note:* Comparison of VAS scores between group A and group B at different time points and VAS scores between group A and group B at different time points at the same time point

**Table 4:** Comparison of Lysholm knee scores between Group A and Group B

Group	Cases	Pre-op	3 months	6 months	1 year	3 years	5 years	*P* value
Group A	7	38.86 ± 3.08	48.86 ± 2.54	71.71 ± 2.69	83.14 ± 3.80	85.71 ± 3.73	90.14 ± 1.68	0.0026
Group B	22	38.59 ± 4.28	49.23 ± 2.78	71.18 ± 3.50	81.95 ± 3.14	87.64 ± 2.19	91.09 ± 1.93	0.001
t		0.15	0.31	0.37	0.83	1.69	1.17	
*P*		0.88	0.76	0.72	0.41	0.1	0.25	

*Note:* Comparison of Lysholm knee joint scores between group A and group B at different time points and Lysholm knee joint scores between group A and group B at different time points at the same time point

## Discussion

DM also known as discoid cartilage, is an anomalous meniscus that manifests as a thick and enlarged disc-shaped malformed cartilage, commonly observed on the lateral side [17]. This condition is characterized by a deficiency in blood supply, which can restrict knee joint mobility and hinder efficient load distribution [18]. Clinical indicators of DM include joint rebound, joint pain, tenderness in the joint space, joint catching, and restricted flexion and extension. The prevalence of lateral DM is notably higher among Asians compared to Western populations. Studies have shown significant variation in incidence rates across different regions and ethnicities [19].

Our study monitored all patients for a minimum of 5 years post-operation using the Ikeuchi grade, VAS scores, and the Lysholm knee scoring scale [20]. We observed significant improvements in both VAS and Lysholm scores within each group over different time periods (*P* < 0.05). However, there were no significant differences in these scores between Group A and Group B at the same stages (*P* > 0.05). These results indicate that arthroscopic discoid meniscoplasty effectively relieves pain and improves knee function without recurrence of symptoms within 5 years.

The outcomes for Group A were rated as excellent in 5 instances, good in 1, fair in 1, with no poor cases, resulting in an 85.71% combined rate of excellent and good outcomes. In Group B, there were 18 excellent outcomes, 3 good outcomes, and 1 fair outcome, with no poor cases, leading to a 95.45% combined rate of excellent and good outcomes. Significant reductions in VAS scores were observed in both groups post-surgery, with no significant differences between the groups at the same stages. Similarly, significant improvements in Lysholm scores were noted in both groups post-surgery, with no significant differences between the groups at the same stages.

Our findings support that arthroscopic discoid meniscoplasty, with or without additional repair and suturing, is a safe and effective surgical method for managing DM. The procedure significantly reduces pain and improves knee joint function, with no recurrence of symptoms over a 5-year follow-up period. Given the higher susceptibility of DM to tears and functional alterations, early surgical intervention is crucial for symptomatic cases to prevent long-term complications [10].

The study has a limited sample size and detects variations in the postoperative rehabilitation procedures among the two groups. The outcomes may be influenced by patients’ adherence and post-discharge rehabilitation status. Factors like the physical activity or work demands of postoperative patients, the intensity of their routines, and negative lifestyle choices like smoking and drinking may impact the recovery of knee joint function to varying extents. Worthwhile mentioning is the absence of a structured categorization for patients based on their work nature, sports interests, and other related factors, indicating the necessity for their inclusion in forthcoming research endeavors. Furthermore, exploring the efficacy of this approach on diverse categories of professional athletes stands out as a promising future research avenue.

## Conclusion

In conclusion, arthroscopic discoid meniscoplasty provides significant pain relief and functional improvement in patients with DM. Both surgical techniques studied (meniscoplasty alone and meniscoplasty with repair and suturing) yield similar outcomes in terms of pain reduction and knee function enhancement. This study highlights the importance of early intervention and tailored surgical approaches based on individual patient conditions and injury severity.

## Data Availability

All data generated or analyzed in this study are included in the present manuscript.
